# Single transmembrane GPCR modulating proteins: neither single nor simple

**DOI:** 10.1093/procel/pwad035

**Published:** 2023-06-14

**Authors:** Meng Wang, Jianjun Lyu, Chao Zhang

**Affiliations:** Department of Plastic and Reconstructive Surgery, Shanghai Ninth People’s Hospital, Shanghai Jiao Tong University School of Medicine, Shanghai 200011, China; Hubei Topgene Research Institute of Hubei Topgene Biotechnology Co., Ltd, East Lake High-Tech Development Zone, Wuhan 430205, China; Fundamental Research Center, Shanghai Yangzhi Rehabilitation Hospital (Shanghai Sunshine Rehabilitation Center), Tongji University, Shanghai 200092, China

The discovery of G-protein coupled receptor (GPCR) accessory proteins has fundamentally redefined the pharmacological concept of GPCR signaling, demonstrating a more complex molecular basis for receptor specificity on the plasma membrane and impressionable downstream intracellular cascades. GPCR accessory proteins not only contribute to the proper folding and trafficking of receptors but also exhibit selectable receptor preferences. The melanocortin receptor accessory proteins (MRAP1 and MRAP2) as well as receptor activity-modifying proteins (RAMPs) are two well-known single transmembrane partners for the regulation of the melanocortin receptors (MC1R–MC5R) and the glucagon receptor (GCGR), respectively. Especially, the MRAP family participates in the pathological control of multiple endocrine disorders and RAMPs contribute to the endogenous regulation of glucose homeostasis. However, the precise mechanisms by which the MRAP and RAMP proteins regulate receptor signaling at atomic resolution remain unknown. Recent progress made in the determination of RAMP2-bound GCGR complexes published on *Cell* unraveled the importance of RAMP2 for the promotion of extracellular receptor dynamics leading to cytoplasmic surface inactivation. Moreover, the new findings on *Cell Research* of the adrenocorticotropic hormone (ACTH)-bound MC2R–Gs–MRAP1 complex disclosed the essential role of MRAP1 for MC2R activation and specificity of ligand recognition. In this article, we reviewed a series of key findings of MRAP proteins in the last decade, the recent structural investigation of the MRAP–MC2R and RAMP–GCGR functional complex, and the expanded identification of new GPCR partners of MRAP proteins. An in-depth understanding of GPCR modulation by single transmembrane accessory proteins will provide valuable insights for the therapeutic drug development to treat multiple GPCR-associated human disorders.

## Introduction

The G-protein coupled receptor (GPCR)-mediated signaling participates in almost all known physiological processes and GPCRs have been validated as a key target family for drug development. GPCRs were originally identified as monomeric seven-transmembrane proteins. Increasing knowledge realized that homo- or hetero-dimerization of GPCR was also essential for receptor transportation, ligand response, and pharmacological cascades. Recent studies have demonstrated that GPCRs could interact with a variety of single transmembrane proteins and these “accessory” proteins exert an indispensable role to ensure accurate folding and proper translocation of functional GPCR receptors to the cell surface. More importantly, these accessory proteins could also affect ligand-induced pharmacology by altering the conformational dynamics of GPCR partners. Notable examples are the family of receptor activity-modifying proteins (RAMPs) and melanocortin receptor accessory proteins (MRAPs).

The RAMP family consists of three members: RAMP1, RAMP2, and RAMP3. It possesses a long extracellular N-terminal (~150 amino acids), a conserved transmembrane structural domain, and a short intracellular C-terminal (~9 amino acids) (McLatchie et al., 1998). RAMPs were originally found to interact with calcitonin-like receptors (CLRs) and required for CLR translocation from the endoplasmic reticulum (ER) to the cell surface (Poyner et al., 2002). Independent screening approaches (BRET (bioluminescence resonance energy transfer) and SBA (suspension bead array)) were recently performed to efficiently expand the list of RAMP-interacting GPCRs from 11 to 44 receptors including Class A, B, C, and adhesion families (Lorenzen et al., 2019; Serafin et al., 2020). To date, studies on RAMP–GPCR interactions are focusing on the glucagon receptor (GCGR) family (Shao et al., 2022). Previous reports have comprehensively elucidated the pharmacology, physiology, and conformational dynamics of RAMP–GPCR complexes (Serafin et al., 2020; Kotliar et al., 2023). Here, we highlighted the new evidence supporting the nature of the interface between RAMP and GPCR chaperones and provided new scope for the development of new drugs and the forecast of how to modulate endogenous GPCR signaling via RAMPs in the future.

The MRAP family comprises two accessory proteins that generate distinct phenotypes by regulating different melanocortin receptors *in vivo*. MRAP1 was originally identified as an accessory partner for maintaining proper translocation and adrenocorticotropic hormone (ACTH) stimulation of MC2R signaling for steroidogenesis in the adrenal gland (Metherell et al., 2005; Roy et al., 2007). MRAP2 was subsequently discovered in 2009 from a cDNA panel of human tissues (Chan et al., 2009). As a single transmembrane protein, MRAP1 and MRAP2 both interact with all five melanocortin receptors and modulate their cell surface expression and ligand-responsive properties. In addition, MRAP2 could form a heterodimer with MRAP1 and similarly increase the membrane surface level of MC2R as MRAP1 (Chan et al., 2009). The expression of MRAP proteins in other tissues besides adrenal and the phenotypic variations between MRAP/MRAP2 knockout and MC2R/MC4R knockout animals attracted much attention. Several studies have demonstrated that MRAP proteins contribute to whole-body energy homeostasis by regulating the physiological actions of non-melanocortin receptors such as prokineticin receptor 1 (PKR1) and ghrelin receptor (GHSR1a) (Chaly et al., 2016; Srisai et al., 2017). Here, we reviewed these key findings of MRAP function and highlighted the most recent discoveries and future directions of MRAP2–GPCR signaling.

## Characterization and physiological functions of the MRAP family

### The particular dual topology

Remarkably, MRAP2 and MRAP1 exhibit functional discrepancies even though they are homologous pairs. Prior to the discovery of MRAP2, MRAP1 was confirmed to form a particular topology of antiparallel homodimers (Sebag and Hinkle, 2007) and even higher-order oligomers in parallel orientation (Chen et al., 2020). MRAP2 could also form heterodimers with MRAP1 (Sebag and Hinkle, 2010). Although MRAP2 assisted MC2R trafficking like MRAP1, it did not promote effective MC2R signaling alone. More evidence found that the molecular ratios of MRAP1 and MRAP2 could also determine the EC50 of MC2R in response to ACTH activation. In our previous studies, we elucidated the internal symmetry of MRAP2 dimers. While the inversion of the N-terminal, C-terminal, or transmembrane regions of MRAP2 did not affect dimer formation, it altered the pharmacological regulation of MC4R signaling (Wang et al., 2021). And we also demonstrated that the complete reversion of the whole MRAP2 protein sequence could generate a functional novel pharmacological modulator of MC4R (Xu et al., 2022).

### MRAP1 in familial glucocorticoid deficiency

Since 2005, more than a dozen of MRAP1 mutations have been discovered to be associated with familial glucocorticoid deficiency (FGD). Twenty percent of Type 2 FGD (FGD2; OMIM: 609196) cases carried MRAP mutations, which could lead to earlier disease onset than FGD1 (Metherell et al., 2005; Jain et al., 2011). In contrast to the majority of missense mutations of MC2R, MRAP1 mutations are typically pre-mRNA splicing or nonsense mutations, which could result in truncated proteins lacking the transmembrane domain, leading to a significant reduction of receptor function (Chung et al., 2010).

The *Mrap1*^−/−^ mouse model, which mimics FGD2 patients, is comparable to *Mc2r* null mice with notable glucocorticoid-deficient ACTH resistance (Chida et al., 2007; Novoselova et al., 2018). However, *Mrap1*^−/−^ mice are deficient in mineralocorticoids and catecholamines, differing from the *Mc2r*^−/−^ mice, which exhibit low levels of aldosterone and catecholamines. Moreover, *Mrap1*^−/−^ animals show substantially reduced adrenal size postnatally and disrupted cortical zonation and progenitor cell differentiation, suggesting an essential role of MRAP1 in adrenal maintenance, self-renewal, and zonation regulation.

### MRAP2 in obesity model

Subsequent studies have mainly focused on the roles of MRAP2 in modulating the physiological functions of the adrenal gland. Up till 2013, two independent back-to-back studies published in *Science* elucidated the physiological roles of MRAP2 in the central nervous system to regulate energy balance, both in embryonic stages and adulthood (Asai et al., 2013; Sebag et al., 2013). Asai et al. observed an obese phenotype in both global and hypothalamic conditional MRAP2 knockout mice. They demonstrated that this obesity syndrome partially resulted from altering centrally expressed MC4R signaling. In support of this statement, another study by Sebag et al. characterized two zebrafish MRAP2 paralogs, MRAP2a and MRAP2b, that differentially altered feeding and somatic growth through the central control of MC4R signaling (Sebag et al., 2013). MRAP2a and Agouti-related protein (AgRP) synergistically inhibit the constitutive activity and ligand-induced signaling of MC4R, thereby maximizing the somatic growth of larval zebrafish. While MRAP2b subsequently expressed in adult zebrafish, converted MC4R from constitutively active to a ligand-dependent receptor by reducing the constitutive activity and increasing the sensitivity to α-MSH stimulation (Sebag et al., 2013).

MRAP2 is clinically susceptible to rare pathogenic mutations. In 2013, Asai et al. first screened the genomic regions of MRAP2 in obese individuals and control cohorts and identified an MRAP2 heterozygous variant locus (E24X) in a patient with a body mass index (BMI) of 63. This finding provided clinical evidence that MRAP2 mutations contributed to severe human obesity (Asai et al., 2013). By performing a large-scale sequencing of MRAP2 in 9,418 individuals, Baron et al. reported a total of 23 rare heterozygous variants associated with an increased risk of obesity (Baron et al., 2019). It was notable that six variants significantly reduced cAMP-PKA (cyclic adenosine monophosphate-protein kinase A) signaling downstream of MC4R to its natural agonist α-melanocyte stimulating hormone (α-MSH). Meanwhile, seven loss-of-function MRAP2 variants (including the above six loci) were responsible for monogenic hyperphagia obesity, hyperglycemia, and hypertension. These multi-metabolic effects might be ascribed to the failure of proper activation of different MRAP2-GPCR pairs in various organs, including the hypothalamus and pancreatic islets.

The clinical study of Baron et al. also evaluated these mutant loci from an evolutionary perspective (Baron et al., 2019). Loss of function of MRAP2 occurred at highly homologous residues in the N-terminal and transmembrane domain, the more conserved region in the chordate species, whereas the more heterogeneous C-terminal functional deletions all occurred at nonconserved positions. These findings reflected the cross-species divergence of the primate nature of MRAP2 functions. Furthermore, fragment deletions and code-shifting mutations of MRAP2 were found to be associated with increased BMI. However, the functionally essential regions of MRAP2 for proper binding to certain metabolic-related GPCRs are still unclear.

## The new exploration of the MRAP family

### Evolutionary selection

The above investigations elicited two aspects of the molecular functions of MRAP2. On one hand, due to high evolutionary selection, the absence or duplicated homologs of melanocortin members occurs in certain species, such as lacking MC3R in tilapia while two MC5R isoforms and two MRAP2 paralogs exist in zebrafish (Sebag et al., 2013; Zhu et al., 2018). Therefore, a group of laboratories further investigated the preservation of MRAP2 in controlling MC4R signaling from the evolutionary perspective (Zhang et al., 2017; Rao et al., 2019; Zhu et al., 2019; Tai et al., 2021; Wen et al., 2021; Wang et al., 2022d). The most ancient MRAP2 protein in the sea lamprey lacks the full C-terminus (Zhu et al., 2019). The transmembrane region of MRAP2 is highly conserved across all chordate species. The N-segment is less conserved, and the C-terminus is the most variable region which may account for the divergence of MRAP2-mediated melanocortin signaling among species (Baron et al., 2019). The presence of MRAP2 demonstrates a fine control on the MC4R pharmacological activity in various species, which may be physiologically related to the complex alterations in feeding behavior and adaptation to their life histories or nutrient conditions.

### Regulation of MRAPs on non-melanocortin receptors

On the other hand, although MC4R and MRAP2 knockout mice both developed obvious obese phenotypes, the double heterozygous (MC4R^+/−^꞉MRAP2^+/−^) mice were overweight than the heterozygous MC4R (MC4R^+/−^) or MRAP2 (MRAP2^+/−^) mice (Asai et al., 2013). Additionally, MC4R knockout mice considerably increased food intake, while MRAP2 deletion did not alter feeding behavior. Moreover, the broad distribution of MRAP2 transcript across tissues than MC4R indicates additional roles of MRAP2 in other systems (Asai et al., 2013; Liang et al., 2018). Therefore, whether the MRAP family could regulate other GPCR signaling attracted much attention for all melanocortin communities. A few evidence have shown that MRAP proteins could regulate the signaling and activity of additional non-melanocortin GPCRs, such as orexin receptor 1 (OX1R), GHSR1a, PKR1, somatostatin receptors (SSTRs), and melanin-concentrating hormone receptor 1 (MCHR1) (Chaly et al., 2016; Rouault et al., 2017; Srisai et al., 2017; Wang et al., 2022b, 2022c). It is noteworthy that these few reported GPCRs share similarities in terms of expressional distribution with MRAP2. In another study, we identified a reciprocal regulation of MC3R and MC4R signaling in the presence of multiple hypothalamic-abundant GPCR partners (Li et al., 2021). Taken together, these findings suggest that MRAP2 may act as a functional link in hypothalamic neurons to control energy homeostasis by synergistically modulating a large group of GPCR networks.

**Figure 1.**
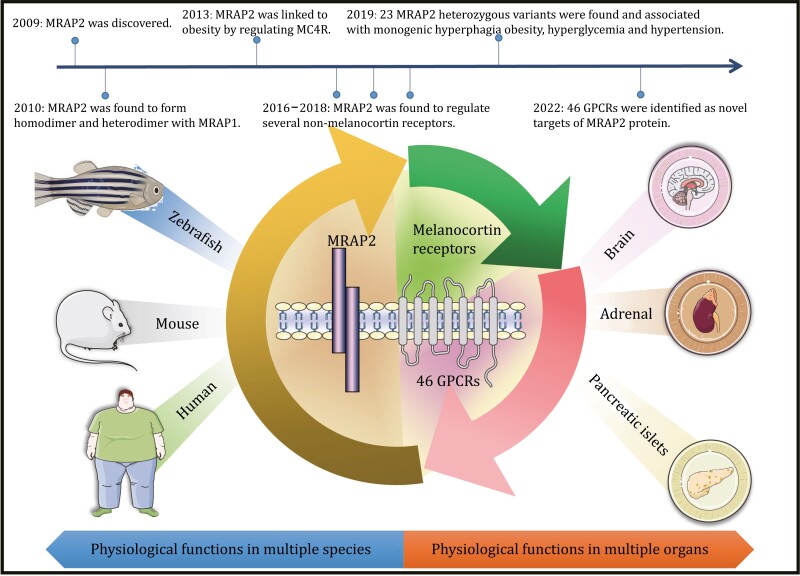
**Exploration of the physiological functions and timelines of MRAP2 in different species and organs since its discovery**.

Thirteen years passed since MRAP2 was discovered and its function had been continuously refined. Benefiting from the rapid development of multi-omics technologies, we first acquired numerous metabolic-related GPCRs that coexpressed with MRAP2 in the same neurons via single-cell RNA sequencing (scRNA-seq). Next, we experimentally screened the majority of GPCRs and found that they not only exhibited direct interactions with MRAP2 proteins but also pharmacologically regulated by MRAP2 in terms of cell surface expression and ligand-induced downstream cascades (Wang et al., 2022a). This work redefined MRAP proteins as broad-spectrum modulators of metabolic-related GPCR signaling *in vitro* and *in vivo* (Fig. 1).

## The structural insight of ligand recognition and GPCR activation by MRAP and RAMP family

Recent advances in structural biology provided important insights into the variable mechanism of accessory protein interaction and GPCR activation. MRAP1 has recently been shown to interact with the ACTH-bound MC2R complex through three major interfaces, in which two with the MC2R and one with the C-terminus of ACTH (Luo et al., 2023). The key interacting sites of MRAP1 with MC2R include an intracellular proximal membrane motif (iJM) and an extracellular proximal membrane motif (eJM). More extensive contact is observed between the eJM of MRAP1 and the extracellular interface of MC2R between the N-terminal end and ECL3. Subsequent alanine mutation analysis determines the more important role of the extracellular interface of MRAP1, while the cytoplasmic interface of MRAP1 is not that essential in the regulation of MC2R signaling. Furthermore, S19 and Y250 of MC2R are the key residues responsible for regulation by MRAP1. MRAP1 functions as a molecular security belt as the tight interaction between the unique basic motif (KKRR) of ACTH and the LDYL motif of MRAP1 ensures the proper binding of ACTH peptide. In particular, the Y20 mutation of MRAP1 completely eliminates ACTH-induced cAMP accumulation (Luo et al., 2023).

**Figure 2.**
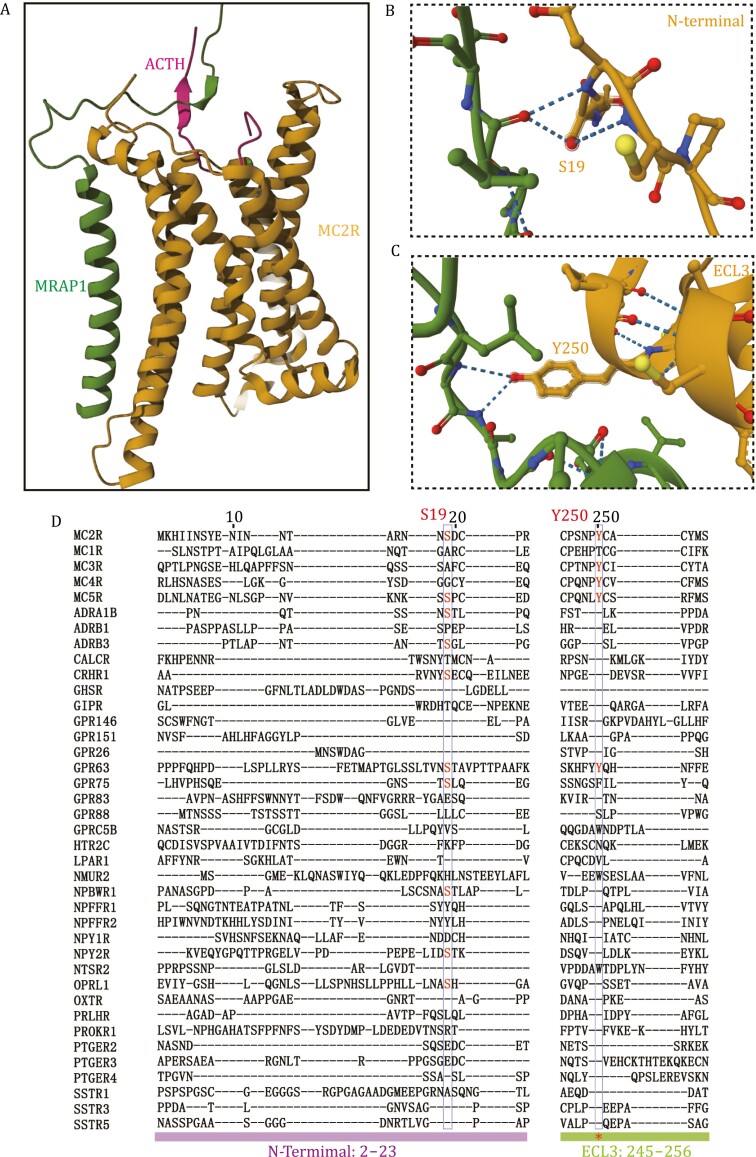
**The key MRAP1-MC2R binding sites.** (A) Ribbon model of the ACTH–MC2R–MRAP1 complex. MRAP1 interacts with the extracellular interface of the receptor and the agonist ACTH to secure specific binding of the ligand (PDB:8GY7). (B and C) Expanded views of the two MC2R-binding sites (B: S19; C: Y250) in the MRAP1-bound MC2R complex. (D) Sequence alignment of melanocortin receptors and other GPCRs interacting with MRAP1. Residues involved in MRAP1 regulation on MC2R are highlighted.

These findings present us an opportunity to explore the influence of MRAP1 on other GPCR activation. We performed a protein alignment analysis and found that the S19 and Y250 in MC2R were not conserved in the newly identified GPCRs that bind to MRAP1 from our study (Fig. 2) (Wang et al., 2022a). This is probably because MRAP1 needs this interface to regulate MC2R activity but not for direct interaction. The complex interacting interface at which diverse GPCRs bind to MRAP1 requires further investigation. Furthermore, our early data suggest that MRAP1 and MRAP2 both assume a dual topology as a functional form when bound to MCRs. However, the stoichiometry of MRAP1 and MC2R was found to be 1:1 in the cryo-EM structure of the ACTH–MC2R–Gs–MRAP1 complex. This cannot exclude the possibility of the existence of a homodimeric MRAP1, as the MRAP1 dimer binding site could be disrupted during the purification. Therefore, the assembly state of MRAP1 and its relevance to functional regulation still need to be further addressed.

As another GPCR accessory protein, the RAMP family has recently attracted attention for interacting and regulating the GCGR family via three classical signaling pathways: Gαs mediated cyclic adenosine monophosphate (cAMP) accumulation, Gαq mediated intracellular Ca^2+^ mobilization and G protein-independent β-arrestin recruitment (Shao et al., 2022). Cryo-EM (cryo-electron microscopy) of RAMP2-GCGR complex demonstrated that RAMP2 transmembrane fragment formed an extended binding interface with TM3 (transmembrane region 3), TM4, and TM5 of GCGR (Krishna Kumar et al., 2023). The interaction induced a broad conformational change of GCGR from the extracellular domain (ECD) to the intracellular surface. RAMP2 selectively regulated the intrinsic conformational heterogeneity of several key regions in GCGR within the ECD and TM6. However, the mechanism of regulation of other GCGRs by RAMP proteins is still unknown. The overlapping distributions of GCGRs and RAMPs, the melanocortin receptors, and MRAPs are seen in all key organs of the metabolic and endocrine system. Clearly, an in-depth investigation of the distribution and physiological relevance of GPCR–RAMP and GPCR–MRAP pairs is needed to comprehend the essential roles of accessory proteins *in vivo*.

## Summary

**Figure 3.**
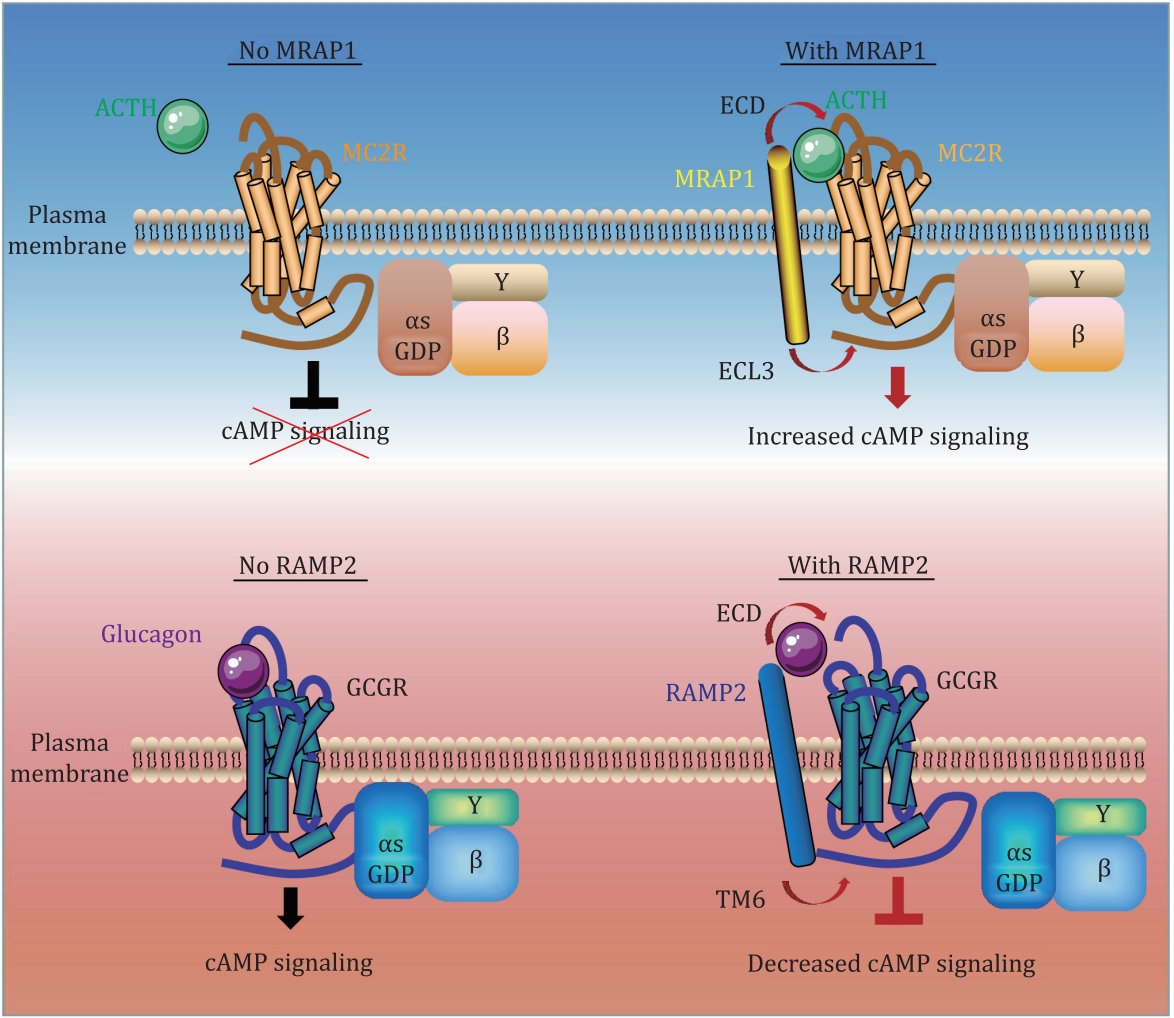
**Schematic illustration of the binding and pharmacological modulation of MRAP1-MC2R and RMAP2-GCGR pairs.** Up panel: Binding of MRAP1 to MC2R and ACTH is a prerequisite for MC2R activation and ACTH-induced cAMP accumulation. Down panel: RAMP2 functions as a negative allosteric regulator of GCGR by enhancing extracellular receptor dynamics and stabilizing the inactive state of the intracellular surface.

Currently, the interface of RAMP2 as a negative allosteric modulator of GCGR and MRAP1 as the positive allosteric modulator of MC2R has been elucidated (Fig. 3). Even these studies have largely expanded the scope with the potential new scientific inspiration of MRAP and RAMP functions, several key aspects of GPCR accessory proteins should be carefully examined in future studies:

1. Among the identified GPCR–MRAP or GPCR–RAMP reciprocal network, endogenous ligands or artificial analogs of several GPCRs have been previously shown to affect the pharmacological responses. Whether MRAP or RAMP influences the effects of these agonists on certain GPCRs *in vivo* needs to be confirmed.
2. Most GPCRs exhibit low transcriptional level due to the sequencing limitation of scRNA-seq technology, which may have missed some important GPCR partners. Thereby, it is crucial to switch to a more effective approach for GPCR mining in the future study. For example, SMART-seq2 or SMART-seq3 with high sequencing depth may be a better approach.
3. Indeed, based on the important role of MRAP2 on MC4R activation, the next step is to resolve the molecular mechanism of MRAP2 on the activation of MC4R signaling. Moreover, are there any common features of the binding sites between GPCR accessory proteins and different GPCRs? It is recommended for researchers to determine the exact interaction mechanism between receptors and RAMPs in the future through several efficient tools, such as cryo-EM and other molecular imaging techniques.
4. It is now increasingly obvious that the physiological axes affected by MRAP and RAMP are quite broad. Especially, the role of MRAP2 in pancreatic islets has become attractive in addition to the adrenal gland and central nervous system. Therefore, what is the function of MRAP2 in other peripheral tissues? Especially, any other organs involved in blood pressure and glucose metabolism, including adipose tissue, the gastrointestinal system, the heart, and kidneys?
5. The implication of GPCR accessory proteins for clinical treatments or drug development of GPCR targets is also worth to be examined. Currently, many GPCRs are implied as anti-tumor targets for immunotherapy (Hauser et al., 2017; Mailankody et al., 2022). Therefore, it would be advantageous for tumor therapy if the surface expression of these GPCRs could be altered by MRAP or RAMP. Particularly, the physiological correlation of MRAP2 and tumors induced by obesity, hypertension, and hyperglycemia is worth to be explored in the future.
